# Supplementary material for: An integrated spatio-temporal view of riverine biodiversity using environmental DNA metabarcoding
Source: Nat Commun. 2024 May 23;15:4372. doi: 10.1038/s41467-024-48640-3 (PMC11116482; doi:10.1038/s41467-024-48640-3)
Supplement: Supplementary file 1 — Supplementary Information [file 41467_2024_48640_MOESM1_ESM.pdf]

Supplementary Information

Supplementary Note 1. Fish life history example: Atlantic salmon and European eel

Adult Atlantic salmon start to return to the river to spawn in the upper reaches of the Conwy in autumn and into the winter, and spawning events in freshwater fish are known to increase the concentration of host eDNA[1–3]. This was reflected in the normalised Atlantic salmon reads in this study which reached their highest levels during in this autumn-winter period. Adult European eels, however, migrate seaward in the autumn to spawn, and the increase in European eel counts in autumn seen in this study is likely due to greater presence in the upper reaches of the Conwy, caused by the culmination of active elver migration back into the river[4], subsequent somatic growth[5] and seeking for winter refuges upstream[6]. It could therefore be a culmination of eDNA being generated from more activity and recruitment, as opposed to just more individuals.

Supplementary Note 2. Annelid life history example: Enchytraeidae

In the case of Enchytraeidae, as many species are associated with terrestrial-aquatic interfaces, includes freshwater, amphibiotic and terrestrial (wet soils)[7,8] forms, it is possible that in winter when a river becomes swollen, these habitats are enveloped, thus releasing a source of eDNA. The greater detection of Lumbriculidae detected at this time of year seen in this study could be due to their tendency to reproduce sexually in later winter[9,10], with gametes providing an additional source of eDNA.

Supplementary Note 3. Nematode life history example: Monhysteridae, Teratocephalidae and Tylenchomorpha

Monhysterids and Teratocephalidae feed on bacteria, detritus and algae which means they can dominate nematode communities in organically enriched sites typical of the decay seen in winter[11,12]. Additionally, laboratory studies on the decomposition processes have shown that Monhysterids adhere to an idiosyncratic diversity model whereby perturbation can cause chaotic biodiversity

patterns through species-specific responses[13], while also having short generation times (two weeks between generations)[14]. All of which demonstrates the potential of Monhysterids to rapidly respond to environmental conditions in the winter with explosions, and subsequent contractions, of cryptic lineages that are governed by bacterial populations, organic matter and abiotic conditions. Teratocephalidae on the other hand are plant pathogens that feed specifically on plant roots using specialized spear-like and straw-sucking mouthparts and could be enriched in winter if more plant material is washed into a river from its banks. Consequently, eDNA derived insights into the diversity of lesser monitored, but ecologically sensitive invertebrates are powerful[15], given the current lack of information on meiofauna in temperate rivers.

Mackerel eDNA transport experiment

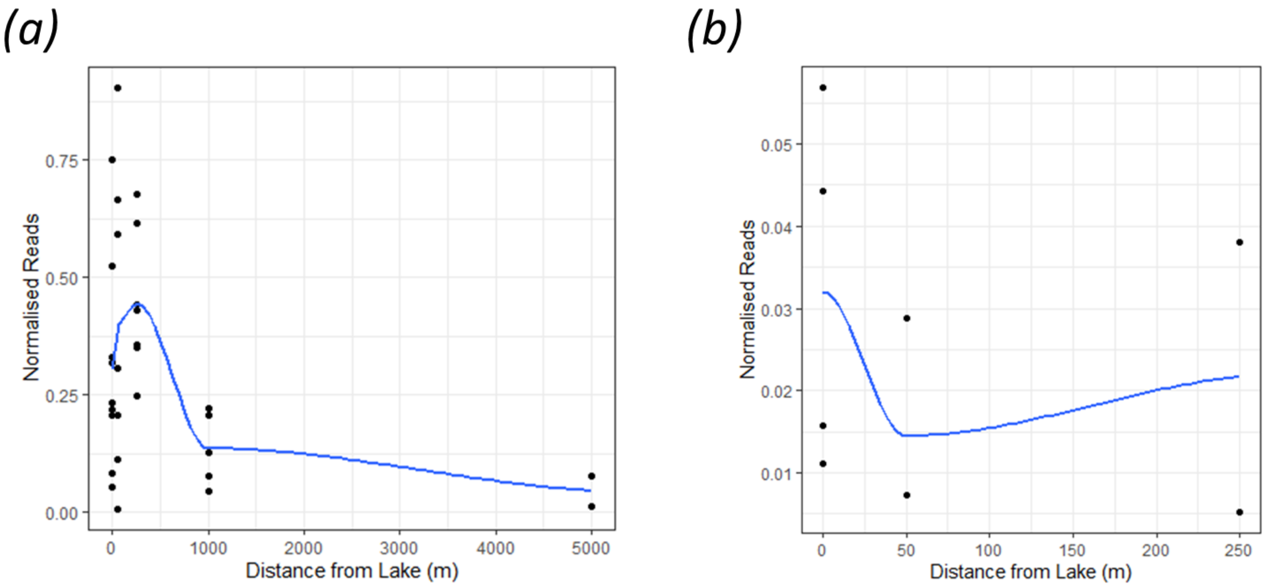

Supplementary Fig. 1 Normalised read counts from the freshly dead Atlantic mackerel (*Scomber scombrus*) introduced at the source of the River Conwy: Llyn Cowy (i.e. 0m distance from the lake). Plots are for reads produced using the (a) 12S and (b) COI marker. Blue line is a fit loess smoothing line.

Supplementary table 1 Information on sampling across the five rivers.

River	Source	Country	Number of sites	Number of timepoints	Sample collection date
Conwy	Llyn Conwy	Wales (UK)	14	19	27th April 2017 - 18th April 2018
Tywi	Llyn Brianne	Wales (UK)	12	1	13th July 2017
Gwash	Rutland Water reservoir	England (UK)	11	1	31st July 2017
Glatt	Greifensee glacial lake	Switzerland	13	1	3rd July 2017
Skaneateles Creek	Skaneateles finger lake	USA	11	1	19th July 2017

Supplementary table 2 Model F statistics and p values from a two-sided analysis of variance assessing the impact of space, time and environmental conditions on alpha diversity in different taxonomic groups. A linear model was used for fish, with fish species count as the response variable, as detected with the 12S marker. A GAM was used for the phyla arthropoda (here, total Arthropoda to delineate from aquatic Arthropoda), Rotifera, Nematoda, Annelida and Mollusca, with the response variable Shannon index, calculated using ASVs detected with the 18S marker. Using the COI marker, arthropod ASVs were further split into aquatic arthropod ASVs.

	Dist. From lake	Season	Days	Dist. From lake: Autumn	Dist. From lake: Spring	Dist. From lake: Summer	Dist. From lake: Winter	pH	Daily flow	Monthly flow	Flow week before sampling	River gradient	Monthly rainfall	Monthly temp.	Conductivity
Fish	**F = 2340.39, p < 0.01 df = 1, 185**	**F = 30.27, p < 0.01 df = 3, 185**	F = 1.54, p = 0.22 df = 1, 185	**F = 6.17, p < 0.01 df = 3, 185**				F = 3.20, p = 0.07 df = 1, 185	F = 1.00, p = 0.32 df = 1, 185	F = 0.04, p = 0.84 df = 1, 185	F = 0.02, p = 0.90 df = 1, 185	**F = 26.81, p < 0.01 df = 1, 185**	F = 2.28, p = 0.13 df = 1, 185	F = 0.59, p = 0.44 df = 1, 185	F = 3.05, p = 0.08 df = 1, 185
Total Arthropoda	**F = 7.68, p < 0.01**	F = 1.53, p = 0.21	F = 1.32, p = 0.26	**F = 4.63, p < 0.01**	**F = 22.24, p < 0.01**	**F = 3.15, p = 0.01**	**F = 10.75, p < 0.01**	**F = 7.08, p = 0.01**	F = 0.14, p = 0.71	**F = 12.58, p < 0.01**	F = 0.14, p = 0.89	F = 0.62, p = 0.43	**F = 3.7, p < 0.01**	F = 2.24, p = 0.08	**F = 3.42, p < 0.01**
Rotifera	F = 0.45, p = 0.53	F = 1.09, p = 0.35	F = 2.68, p = 0.13	F = 0.08, p = 0.8	F = 0.32, p = 0.62	F = 1.54, p = 0.17	F = 0.65, p = 0.47	**F = 6.08, p < 0.01**	F = 0.12, p = 0.72	F = 0.88, p = 0.5	F = 0.05, p = 0.82	**F = 2.23, p = 0.04**	F = 1.11, p = 0.29	F = 1.45, p = 0.24	F = <0.01, p = >0.99
Nematoda	F = 1.69, p = 0.15	**F = 3.13, p = 0.02**	**F = 4.67, p < 0.01**	F = 1.91, p = 0.14	**F = 3.38, p = 0.01**	F = 0.1, p = 0.78	**F = 4.28, p < 0.01**	**F = 10.07, p < 0.01**	**F = 6.56, p = 0.01**	**F = 15.95, p < 0.01**	**F = 11.51, p < 0.01**	**F = 2.8, p = 0.02**	**F = 9.39, p < 0.01**	**F = 9.15, p < 0.01**	**F = 7.97, p = 0.01**
Annelida	**F = 3.45, p = 0.02**	**F = 16.25, p < 0.01**	**F = 7.24, p < 0.01**	F = 0.26, p = 0.65	F = 1.72, p = 0.24	F = 0.94, p = 0.39	F = 1.93, p = 0.07	**F = 5.75, p < 0.01**	F = 3.2, p = 0.08	**F = 6.13, p = 0.01**	**F = 5.34, p = 0.02**	F = 0.92, p = 0.34	F = 2.78, p = 0.1	F = 0.18, p = 0.67	**F = 3.12, p < 0.01**
Mollusca	**F = 7.89, p < 0.01**	F = 2.02, p = 0.12	**F = 2.48, p = 0.02**	-	-	-	-	-	-	-	-	-	-	-	-
Aquatic Arthropoda	**F = 2.17, p = 0.05**	**F = 6.73, p < 0.01**	F = 0.81, p = 0.37	**F = 4.76, p = 0.05**	F = 0.83, p = 0.30	F = 1.27, p = 0.28	F = 1.05, p = 0.38	**F = 4.34, p = 0.04**	F = 1.07, p = 0.30	**F = 9.31, p < 0.01**	-	F = 0.02, p = 0.89	F = 1.83, p = 0.18	**F = 3.86, p < 0.01**	**F = 2.53, p = 0.04**

River conwy enivromental conditions

(a)

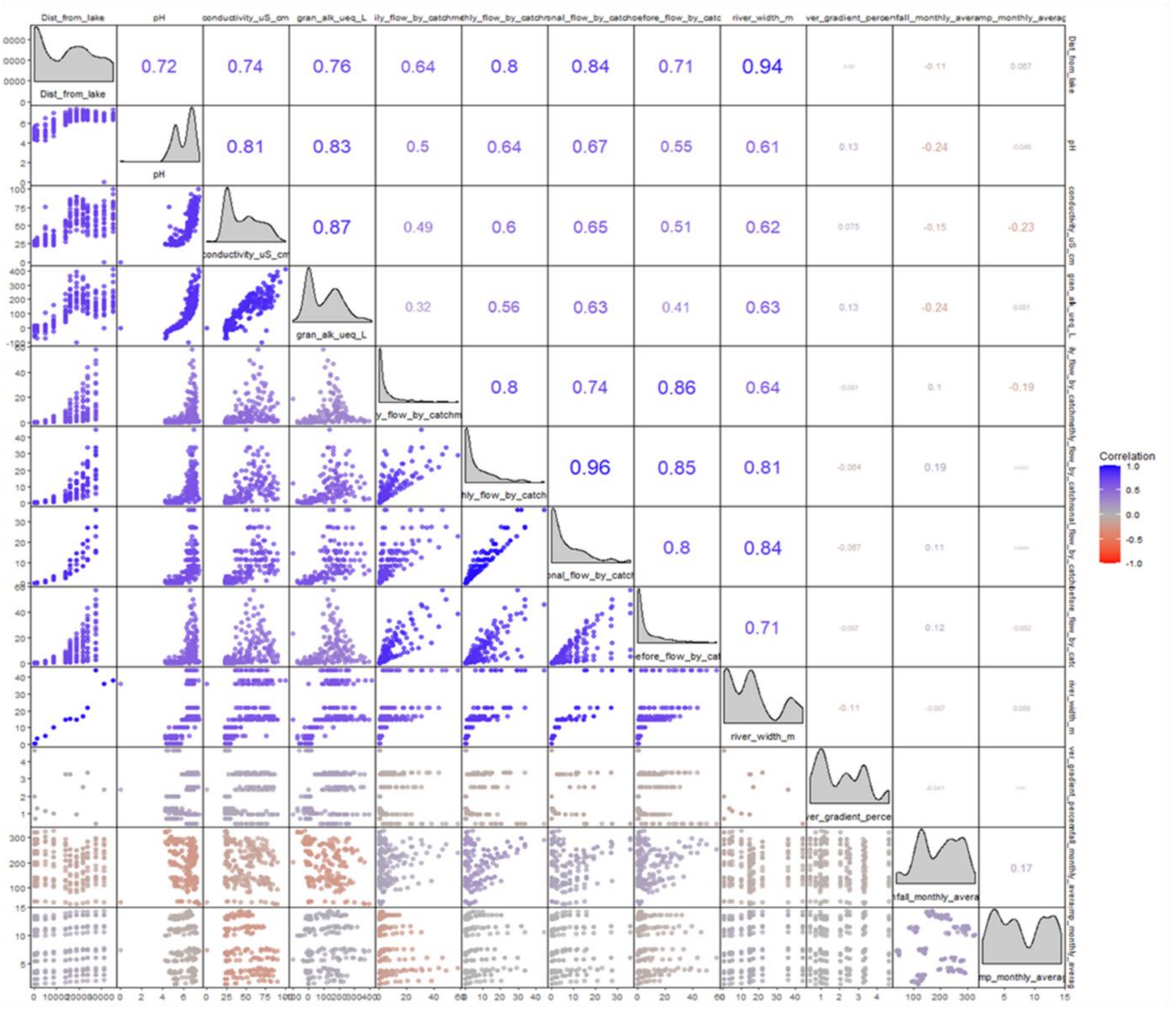

(b)

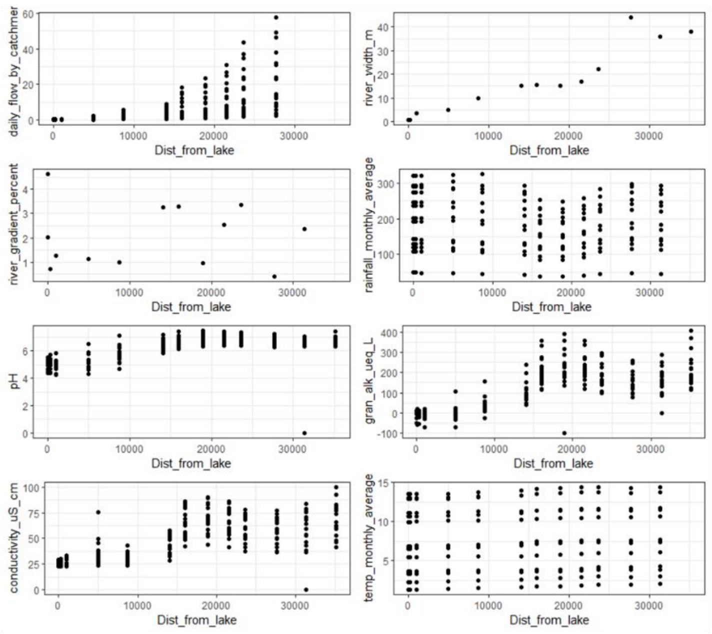

(c)

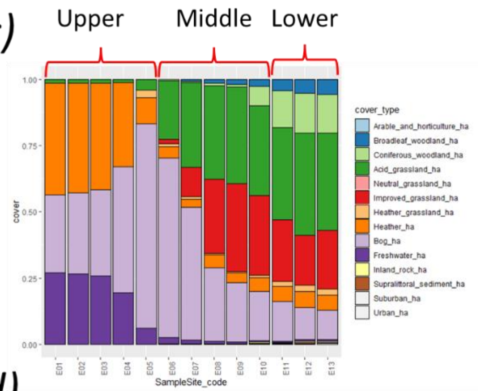

(d)

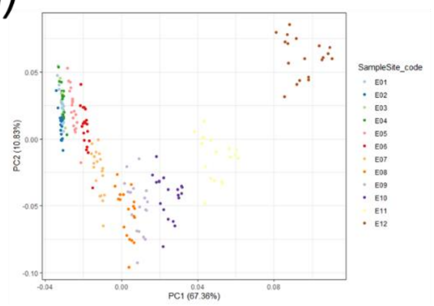

Supplementary figure 2 (a) Co-correlation between environmental conditions along the River Conwy, in addition to (b) environmental metadata across distance from the lake (m) and (c) land use associated with eDNA samples taken from the River Conwy. A (d) principal component analysis combining environmental and land use data is also included, coloured by sample site.

Sparse partial least squares analysis (sPLS)

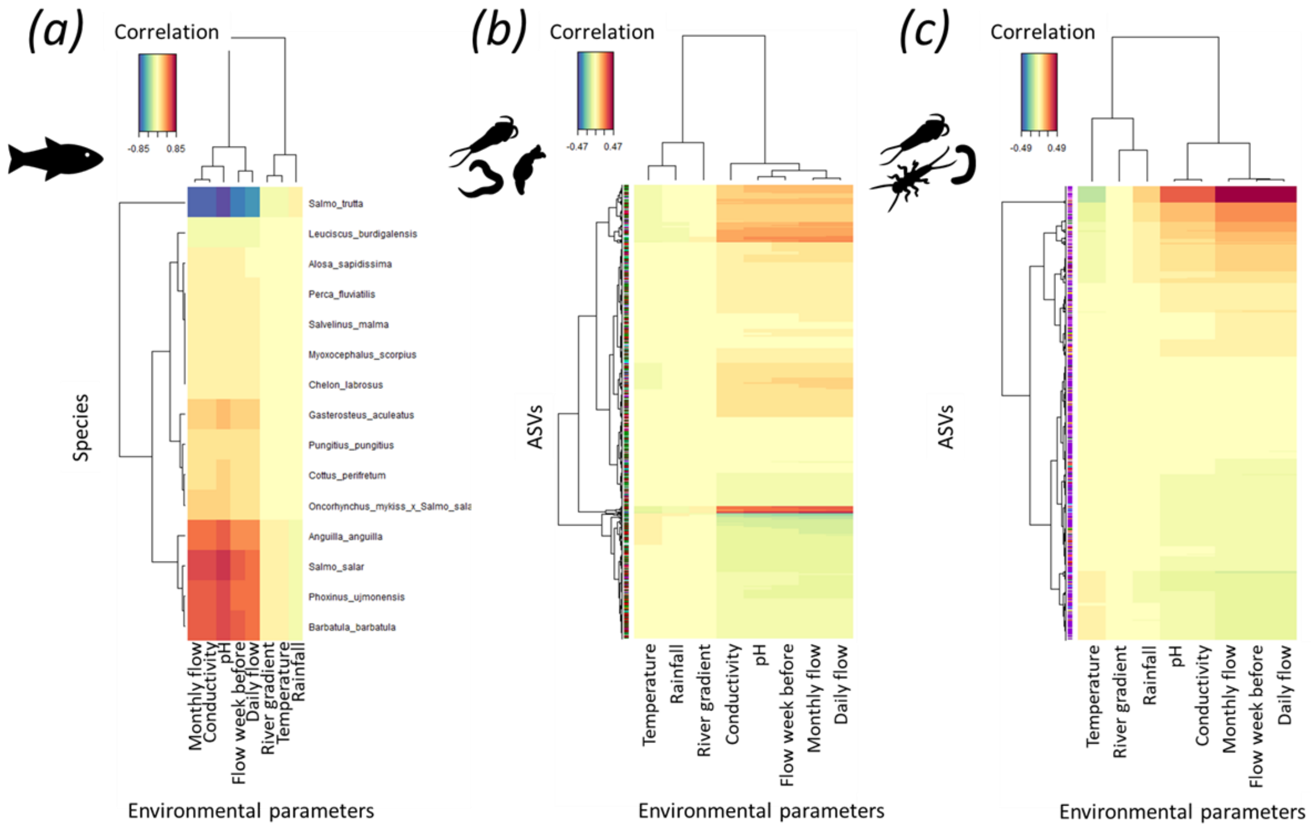

Supplementary figure 3 Clustered heat maps displaying correlations between taxa/ASVs and environmental parameters from the sparse partial least squares analysis (sPLS) for the River Conwy. Included are (a) fish species detected with the 12S marker, (b) metazoan ASVs detected with the 18S marker, and (c) aquatic arthropod ASVs detected with the COI marker. For (b) metazoans and (c) aquatic arthropods, ASV nodes are coloured to represent different phyla and order, respectively. sPLS analysis was conducted using the 'spls' function in the package 'mixOmics'[16] with one component and the canonical algorithm, and results were plotted using the 'cim' function. The brown trout (*Salmo trutta*) was the only species detected by the 12S to show a negative correlation across environmental conditions due to it being the only species present in the first five sites.

Supplementary table 3 Species removed from the 12S analyses in the Conwy, Tywi and Gwash, along with their possible origin.

River	Species	Common name	Possible origin
**Conwy**	*Gadus macrocephalus*	Pacific cod	Human consumption/waste
	Hippoglossus stenolepis	Pacific halibut	Human consumption/waste
	Melanogrammus aeglefinus	Haddock	Human consumption/waste
	Platichthys stellatus	Starry flounder	Human consumption/waste
	Ammodytes personatus	Pacific Sand Lance	Seabirds
	Clupea harengus	Atlantic herring	Seabirds
	Clupea pallasii	Pacific herring	Seabirds
	Copadichromis virginalis	Haplochromine cichlid	Human consumption/waste (tilapia)
	Favonigobius gymnauchen	Sand goby	Seabirds
	Pholis crassispina	Rock gunnel	Seabirds
	Scomber scombrus	Atlantic mackerel	Positive control & human consumption/waste
	Salmo obtusirostris	Adriatic trout	Likely misidentified brown trout
	Anguilla rostra	American eel	Likely misidentified European eel
**Tywi**	*Platichthys stellatus*	Starry flounder	Human consumption/waste
**Gwash**	*Sardina pilchardus*	European pilchard	Human consumption/waste

Supplementary table 4 Outliers removed from beta diversity analysis, as identified by nonmetric multidimensional scaling (NMDS) plots. Colours represent sample sites.

Dataset	Number of samples removed / total number available for analysis after filtering	Samples removed	NMDS plot before and after removal of outliers
12S fish	NA	NA	NA
18S metazoans	6/242	E04_T07<br>E01_T19<br>E02_T01<br>E14_T01<br>E01_T09<br>E01_T18	Before:<br>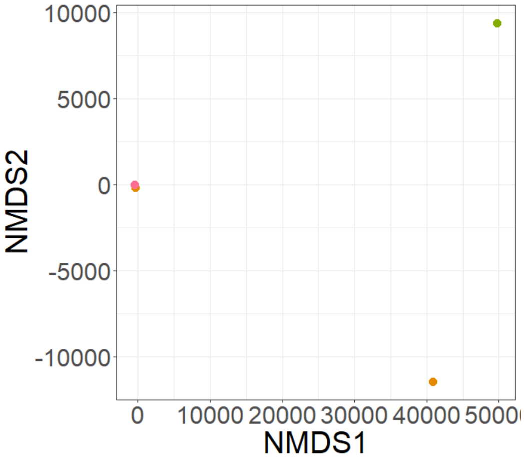<br><br>After:<br>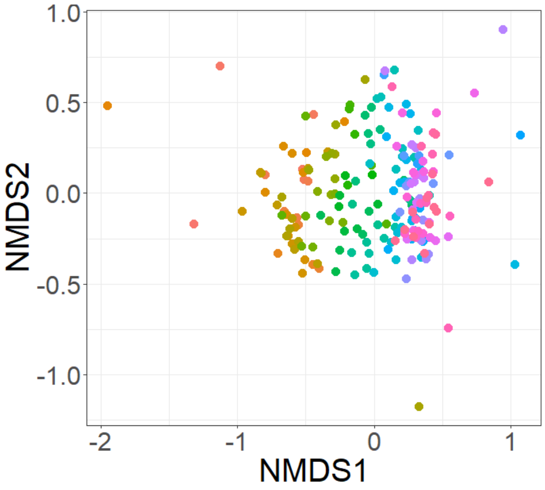
18S arthropods	4/232	E12_T03<br>E05_T10<br>E04_T05<br>E06_T10	Before:

<table>
<tr><td></td><td></td><td></td><td>
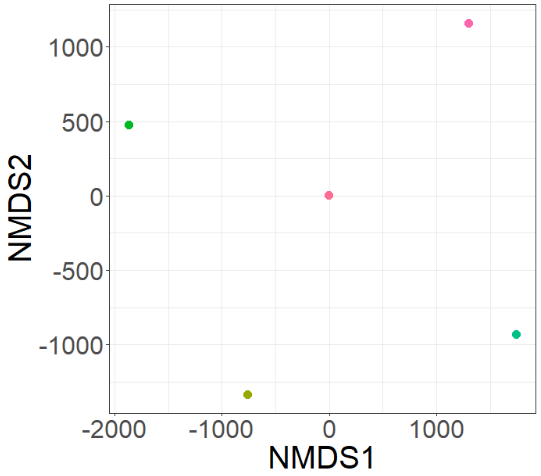

After:
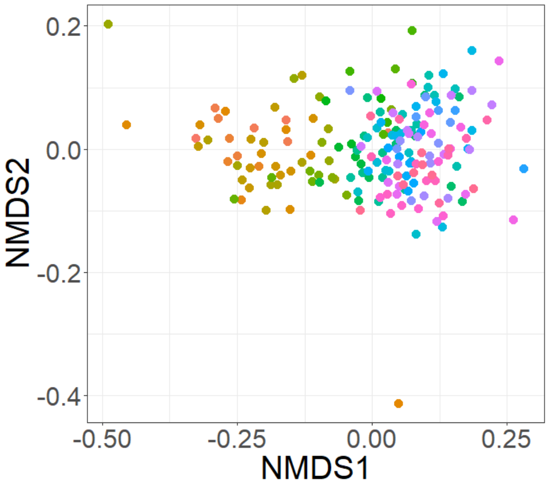

</td></tr>
<tr><td>18S
rotifers</td><td>2/230</td><td>E02_T01
E01_T19</td><td>
Before:
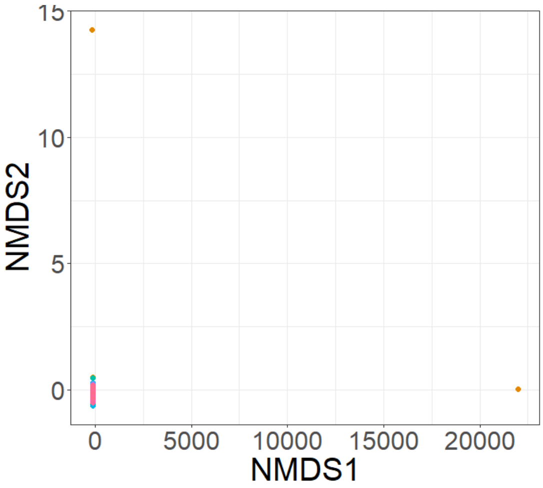

After:
</td></tr>
</table>

			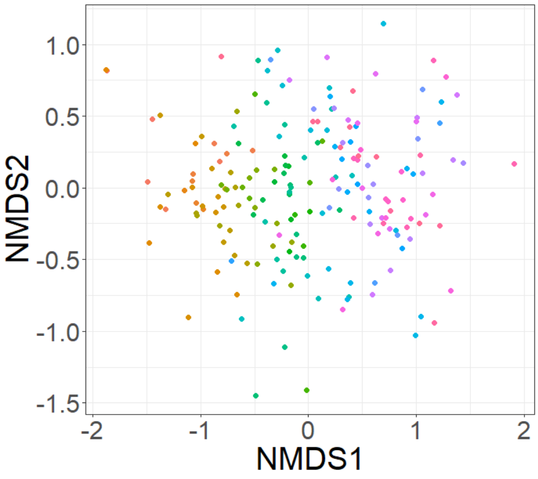
18S molluscs	4/136	E12_T03<br>E05_T10<br>E04_T05<br>E06_T10	Before:<br>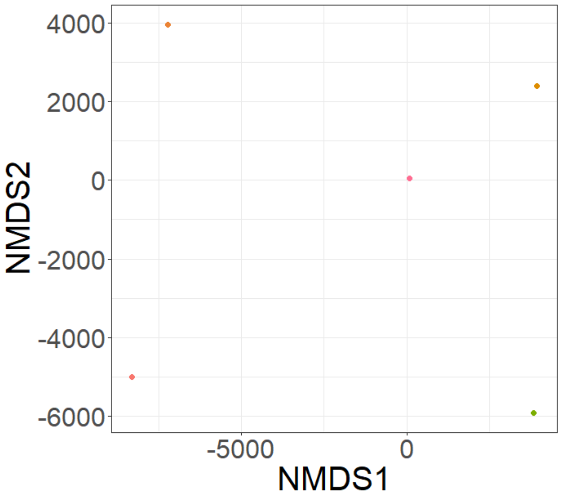<br>After:<br>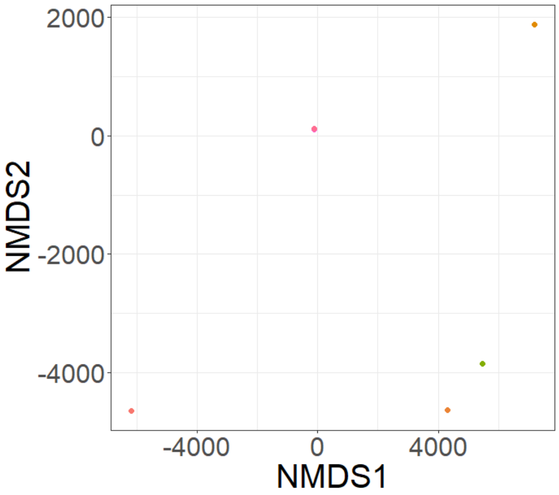
18S nematodes	9/224	E08_T03<br>E10_T04<br>E02_T03<br>E12_T04<br>E12_T08<br>E11_T10<br>E09_T04<br>E08_T02	Before:

		E06_T10	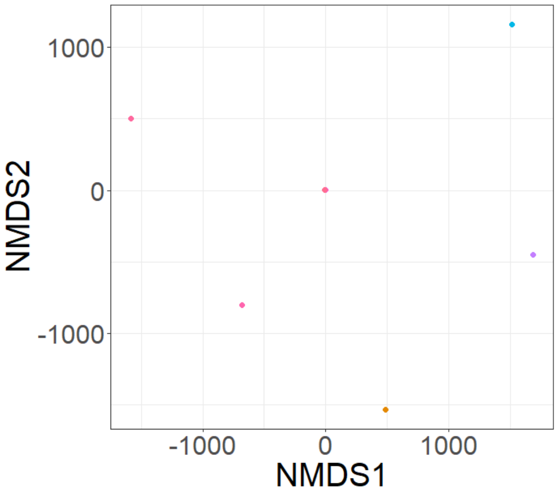<br>After:<br>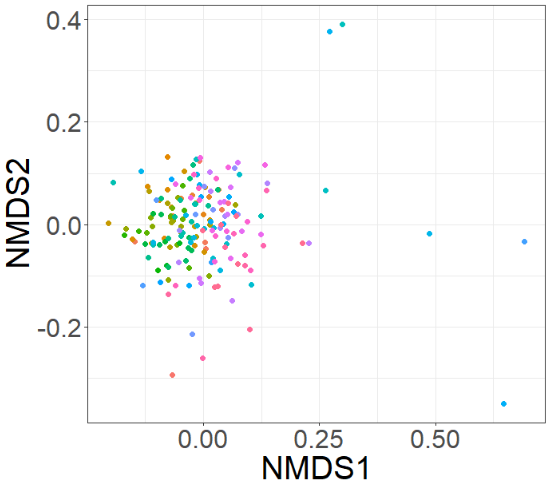
18S annelids	NA	NA	NA
COI aquatic arthropods	50/159	E02_T14<br>E11_T11<br>E03_T04<br>E09_T01<br>E02_T11<br>E07_T07<br>E08_T05<br>E05_T12<br>E05_T18<br>E04_T13<br>E04_T04<br>E03_T15<br>E11_T08<br>E06_T06<br>E06_T11<br>E02_T13<br>E05_T09<br>E05_T03<br>E10_T03<br>E01_T15<br>E10_T05<br>E01_T13<br>E10_T06	Before:<br>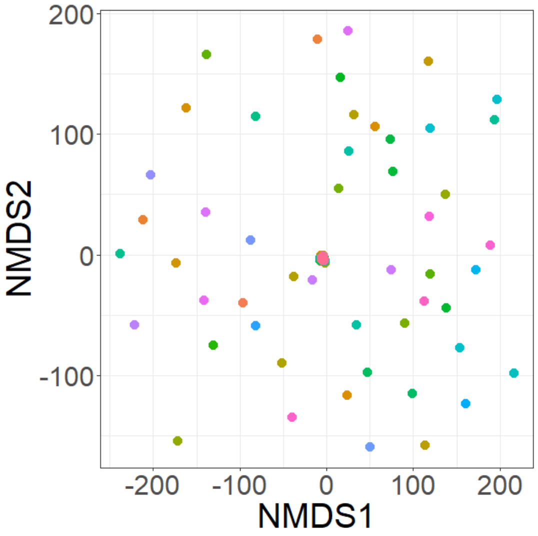<br>After:

		E04_T12<br>E04_T03<br>E03_T13<br>E02_T17<br>E03_T12<br>E01_T09<br>E11_T15<br>E11_T12<br>E09_T11<br>E04_T17<br>E07_T12<br>E05_T08<br>E10_T12<br>E05_T17<br>E03_T14<br>E05_T10<br>E06_T12<br>E09_T15<br>E08_T13<br>E07_T04<br>E10_T15<br>E06_T17<br>E10_T11<br>E09_T12<br>E04_T18<br>E07_T11<br>E06_T15	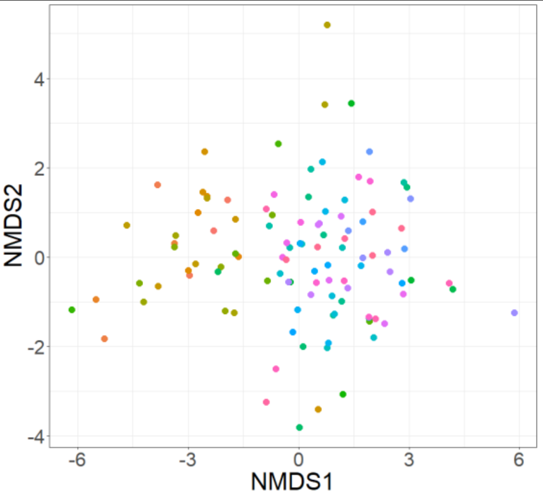

Supplementary table 5 Number of amplicon sequence variants (ASVs) recovered from metabarcoding dataset for each of the loci, both before and after filtering and taxonomy assignment.

**Genetic loci**	**Total ASVs identified**	**ASVs remaining after filtering and taxonomy assignment**
12S	38,287	1,270
18S	162,433	4,526 (metazoan)
COI	126,886	955 (metazoan)

Taxonomic composition of samples in the Glatt, Tywi, Gwash and Skaneateles

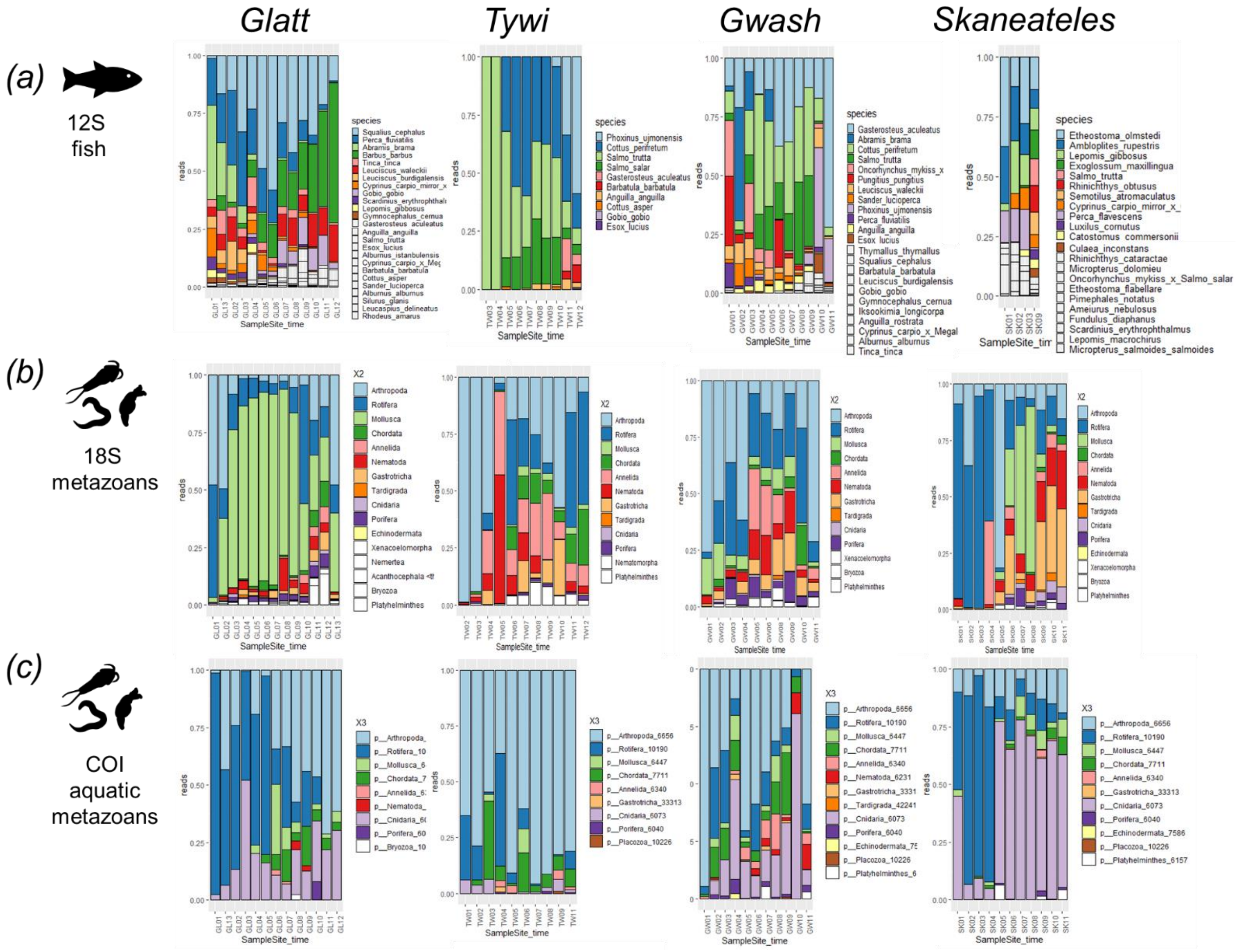

Supplementary figure 4 Stacked bar plots show normalised reads at sites in the rivers Glatt, Tywi, Gwash and Skaneateles Creek, and coloured by taxonomy, with sample sites along the x axis and relative read abundance on the y axis. Stacked bar plots represent (a) fish detected with the 12S marker, (b) metazoans detected with the 18S marker and (c) aquatic arthropods detected with the COI marker. Taxonomic identification is shown at the species level for the 12S marker, and at the phylum level for the 18S and COI markers.

Rarefaction curves

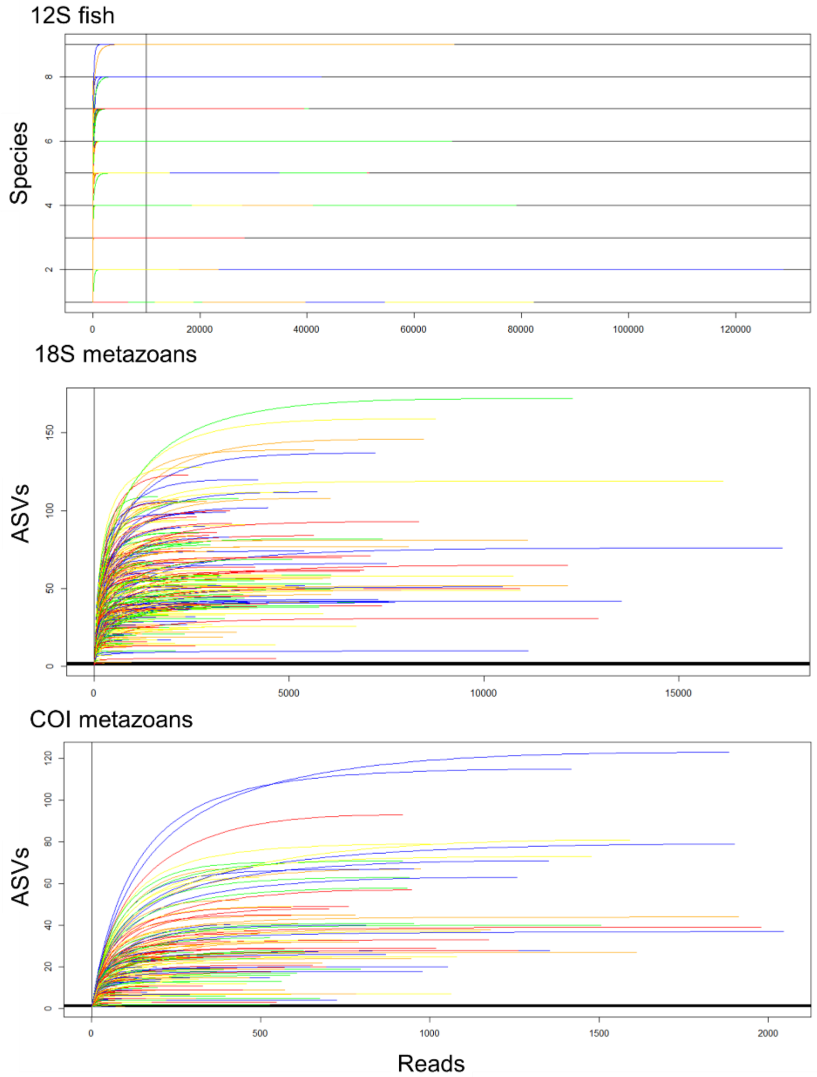

Supplementary figure 5 Rarefaction curves, post filtering process, for the species detected by the 12S marker, as well as the amplicon sequence variants (ASVs) detected using the 18S and COI markers. Colours are randomly assigned and are to help distinguish curves between samples.

Supplementary Discussion

The Conwy dataset was reanalysed using presence/absence, unlike the abundance data used in the main manuscript, to demonstrate the utility of using abundance data, and demonstrate that most of the trends are robust across the different data types:

i) **12S, 18S and COI beta diversity**

There were minimal changes seen between using abundance and presence/absence. For example, the 18S PERMANOVA, out of the 57 combinations of phyla and variables tested, there were only four discrepancies (7%) in significance between the analysis featuring abundance and presence/absence data. Using abundance found significant impacts of environmental variables where presence/absence did not. Although the p values for presence/absence in these cases were not significant, they were not far from it (p value range of those that became insignificant in presence/absence analysis: 0.061 – 0.191).

For COI PERMANOVA, there were no discrepancies in significance between the analysis featuring abundance and presence/absence data.

For the 12S PERMANOVA, out of the 11 variables tested, there were only two discrepancies (18%) in significance between the analysis featuring abundance and presence/absence data. As for the 18S, using abundance found significant impacts of environmental variables where presence/absence did not. A full model output for presence/absence and abundance is provided.

A similar trend between abundance and presence/absence, in terms of importance distance from the lake plays, was seen in the NMDS plots for metazoans detected with the 18S marker, but it is flipped on the x axis (supplementary figure 6). Again, a similar trend between abundance and presence/absence, in terms of importance distance from the lake plays, was seen in the NMDS plots for aquatic arthropods detected using the COI marker (supplementary figure 7).

NMDs plot for metazoans (18S marker)

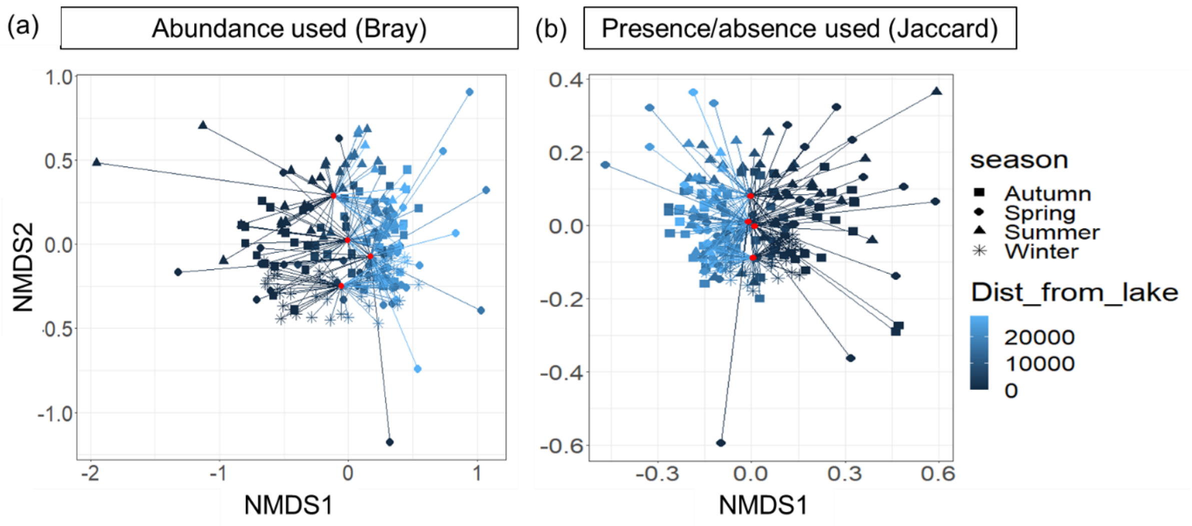

Supplementary figure 6 NMDS plot of River Conwy metazoan ASVs detected with the 18S marker, and whether (a) relative abundance and Bray Curtis dissimilarity were used, or (b) presence absence and Jaccard dissimilarity. Plots coloured by distance the sample was taken from the lake (Llyn Conwy), with shapes denoting season, red dots displaying seasonal mean NMDS scores and lines connecting datapoints with their respective seasonal means.

NMDS plot for aquatic arthropods (COI marker)

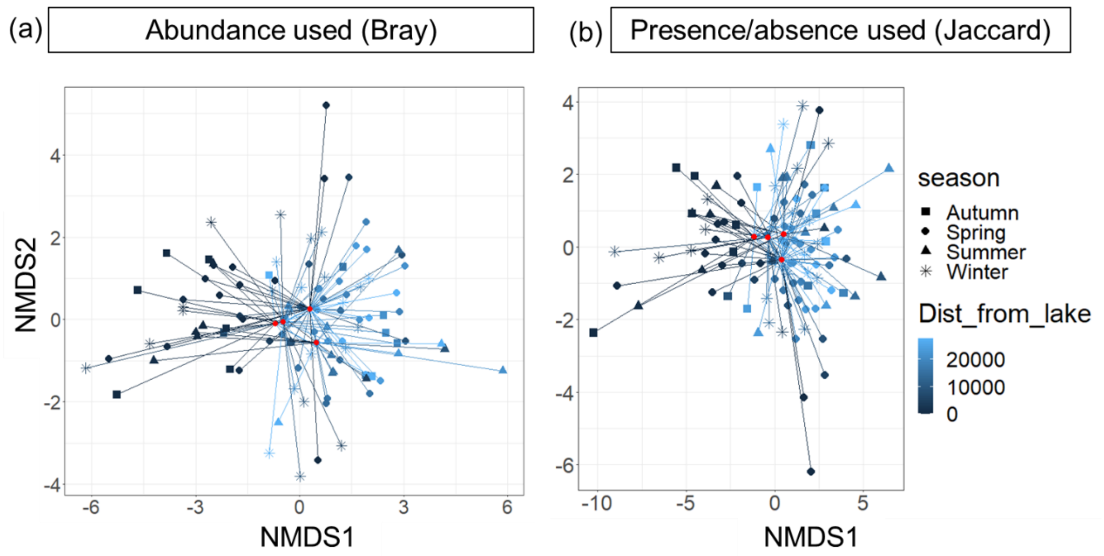

Supplementary figure 7 NMDS plot of River Conwy aquatic arthropod ASVs detected with the COI marker, and whether (a) relative abundance and Bray Curtis dissimilarity were used, or (b) presence

absence and Jaccard dissimilarity. Plots coloured by distance the sample was taken from the lake (Llyn Conwy), with shapes denoting season, red dots displaying seasonal mean NMDS scores and lines connecting datapoints with their respective seasonal means.

When looking at the NMDS plots for fish detected with the 12S marker, using presence/absence data, there is a loss of granularity, with many samples having the same number of species, as presence/absence does not account for species that are at low abundance (supplementary figure 8). However, overall, the trend remains the same.

NMDS for fish (12S marker)

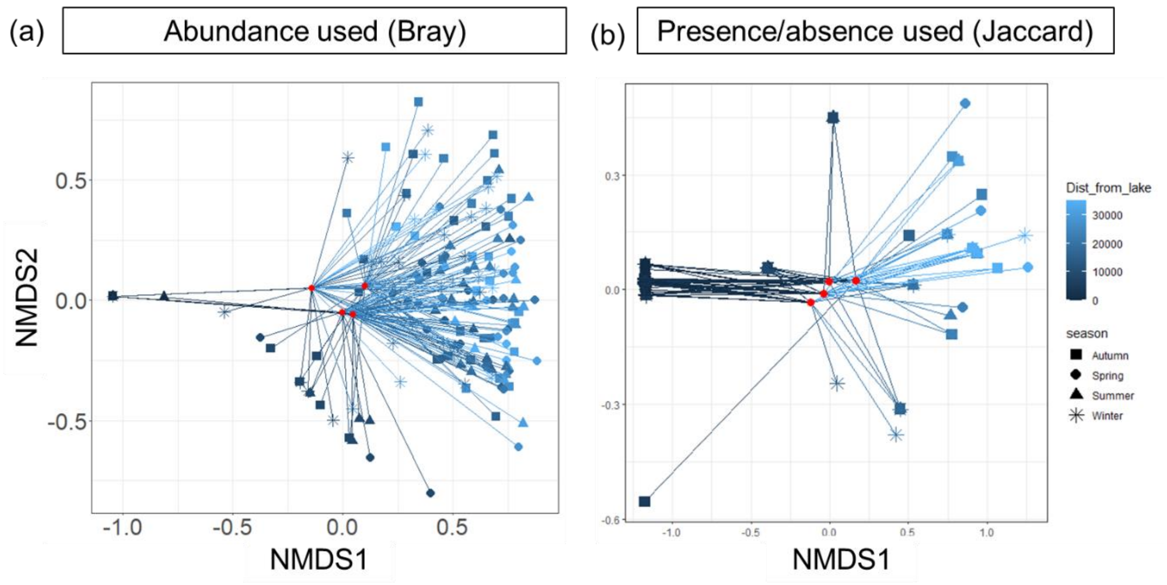

Supplementary figure 8 NMDS plot of River Conwy fish species detected with the 12S marker, and whether (a) relative abundance and Bray Curtis dissimilarity were used, or (b) presence absence and Jaccard dissimilarity. Plots coloured by distance the sample was taken from the lake (Llyn Conwy), with shapes denoting season, red dots displaying seasonal mean NMDS scores and lines connecting datapoints with their respective seasonal means.

ii) 18S and COI alpha diversity

To test the impact of swapping abundance with presence/absence for alpha diversity, instead of using the Shannon index as a measure of alpha diversity, which requires abundances to calculate evenness (the distribution of abundances among species), it was instead replaced with species richness (number of species). Spatiotemporal trends were robust between alpha diversity metrics (abundance and presence/absence) and between markers (18S and COI) with few exceptions (e.g., the presence/absence meant the winter diversity signal was exaggerated in nematodes, and flattened in molluscs) (supplementary figure 9 & 10).

Alpha diversity for metazoans (18S marker) between abundance and presence/absence data sources

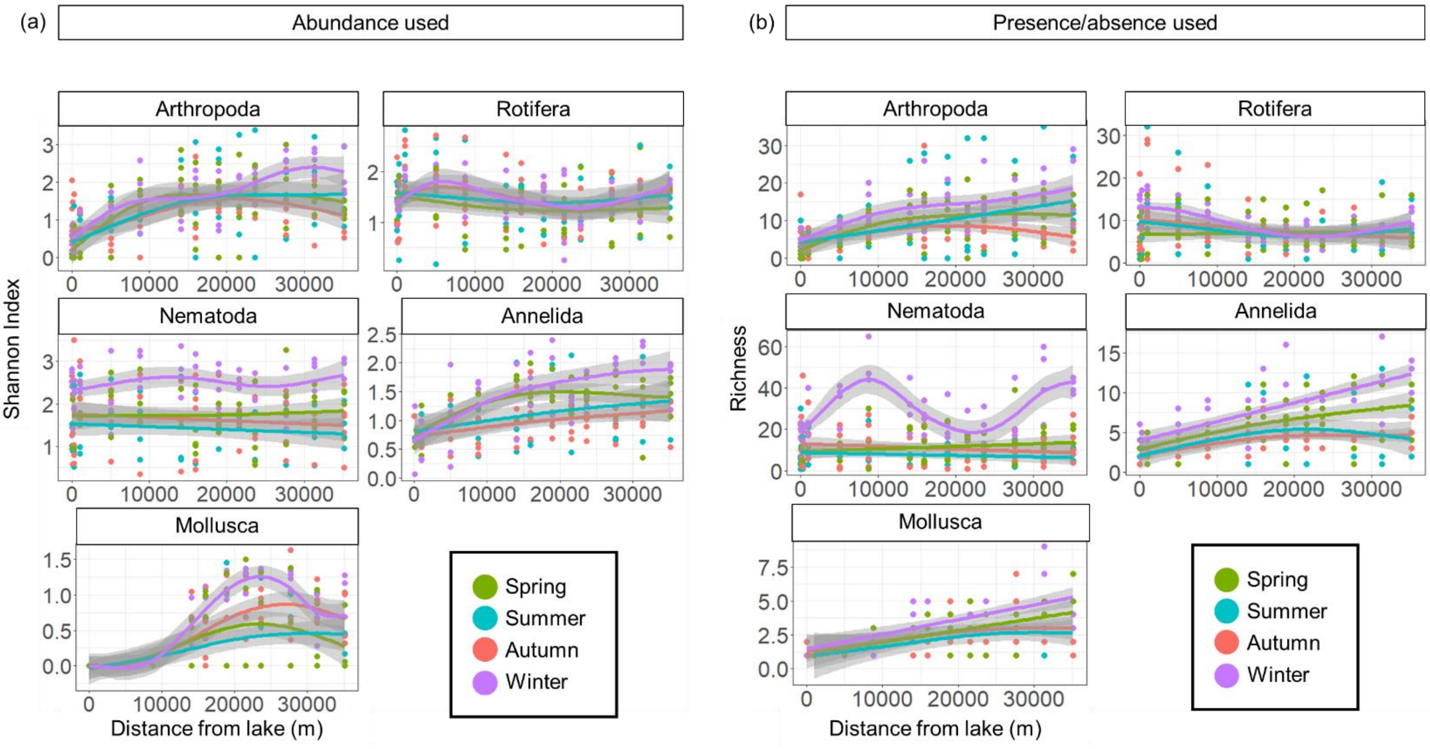

Supplementary figure 9 Plots of (a) Shannon index and (b) richness of ASVs in the most abundant metazoan phyla (annelids, arthropods, nematodes, molluscs and rotifers). Diversity is shown over distance from the lake, coloured by season, with smoothed conditional means and 95% grey confidence intervals provided by generalised additive models.

Alpha diversity for aquatic arthropods (COI marker) between abundance and presence/absence data sources

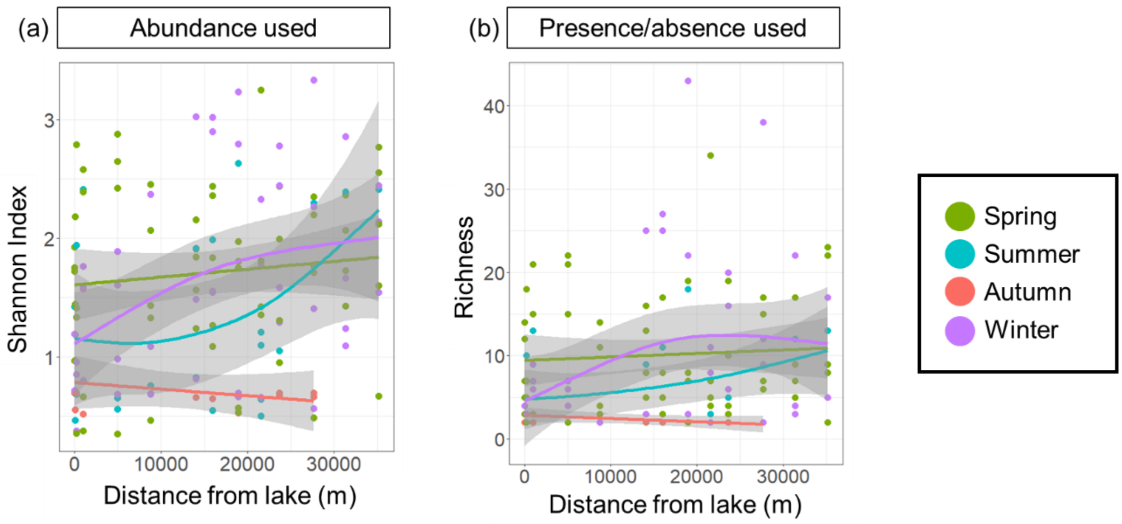

Supplementary figure 10 Plots of (a) Shannon index and (b) richness of aquatic arthropod ASVs. Diversity is shown over distance from the lake, coloured by season, with smoothed conditional means and 95% grey confidence intervals provided by generalised additive models.

Considering other parameters, a larger impact of using presence/absence was seen on alpha diversity when compared to the change seen on beta diversity. Out of the 63 combinations of phyla and variables tested, there were 29 discrepancies (38%) in significance between the analysis featuring abundance and presence/absence data. 20 of these discrepancies occurred as the result of the presence/absence changing non-significant results to significant. This shift is likely due to very rare species now having an equal weighting to common species. We predict that the removal of information on abundance, and thus evenness, means that the effect size of the variables is disproportionately amplified, thus generating more significant effects.

References:

1. Takeuchi, A. *et al.* Release of eDNA by different life history stages and during spawning activities of laboratory-reared Japanese eels for interpretation of oceanic survey data. *Sci. Reports 2019 91* **9**, 1–9 (2019).

2. Tsuji, S. & Shibata, N. Identifying spawning events in fish by observing a spike in environmental DNA concentration after spawning. *Environ. DNA* **3**, 190–199 (2021).

3. Levi, T. *et al.* Environmental DNA for the enumeration and management of Pacific salmon. *Mol. Ecol. Resour.* **19**, 597–608 (2019).

4. Naismith, I. A. & Knights, B. Migrations of elvers and juvenile European eels, Anguilla anguilla L., in the River Thames. *J. Fish Biol.* **33**, 161–175 (1988).

5. White, E. M. & Knights, B. Dynamics of upstream migration of the European eel, Anguilla anguilla (L.), in the Rivers Severn and Avon, England, with special reference to the effects of man-made barriers. *Fish. Manag. Ecol.* **4**, 311–324 (1997).

6. Riley, W. D., Walker, A. M., Bendall, B. & Ives, M. J. Movements of the European eel (Anguilla anguilla) in a chalk stream. *Ecol. Freshw. Fish* **20**, 628–635 (2011).

7. Martínez-Ansemil, E. & Collado, R. Two new species of freshwater Oligochaeta from the North-west Iberian Peninsula: Krenedrilus realis sp. nov. (Tubificidae) and Cernosvitoviella bulboducta sp. nov. (Enchytraeidae). *J. Zool.* **240**, 363–370 (1996).

8. Briones, M. J. I. *et al.* Substrate quality and not dominant plant community determines the vertical distribution and C assimilation of enchytraeids in peatlands. *Funct. Ecol.* **34**, 1280–1290 (2020).

9. White, D. S., Klahr, P. C. & Robbins, J. A. Effects of Temperature and Density on Sediment Reworking by Stylodrilus Heringianus (Oligochaeta: Lumbriculidae). *J. Great Lakes Res.* **13**, 147–156 (1987).

10. Timm, T. Fate of Lamprodrilus isoporus (Oligochaeta: Lumbriculidae) in eutrophic lakes. *Biol.* **71**, 5–15 (2016).

11. De Mesel, I. *et al.* Top-down impact of bacterivorous nematodes on the bacterial community

structure: a microcosm study. *Environ. Microbiol.* **6**, 733–744 (2004).

12. Mulder, C. *et al.* Observational and Simulated Evidence of Ecological Shifts within the Soil Nematode Community of Agroecosystems under Conventional and Organic Farming. *Ecology* **17**, 516–525 (2003).

13. De Mesel, I., Derycke, S., Swings, J., Vincx, M. & Moens, T. Role of nematodes in decomposition processes: Does within-trophic group diversity matter? *Mar. Ecol. Prog. Ser.* **321**, 157–166 (2006).

14. Moens, T. *et al.* Ecology of free-living marine nematodes. in *Handbook of Zoology Volume 2 Nematoda* (ed. Schmidt-Rhaesa, A.) 135 (De Gruyter, 2013).

15. Seymour, M. *et al.* Executing multi-taxa eDNA ecological assessment via traditional metrics and interactive networks. *Sci. Total Environ.* **729**, 138801 (2020).

16. Rohart, F., Gautier, B., Singh, A. & Lê Cao, K. A. mixOmics: An R package for ‘omics feature selection and multiple data integration. *PLOS Comput. Biol.* **13**, e1005752 (2017).
