# Supplementary material for: An integrated spatio-temporal view of riverine biodiversity using environmental DNA metabarcoding
Source: Nat Commun. 2024 May 23;15:4372. doi: 10.1038/s41467-024-48640-3 (PMC11116482; doi:10.1038/s41467-024-48640-3)
Supplement: Supplementary file 3 — Description of Additional Supplementary Files [file 41467_2024_48640_MOESM3_ESM.pdf]

Description of Additional Supplementary Files

File Name: Supplementary Data 1

Description: Metadata associated with each of the samples used in this study, corresponding with the sequences uploaded to the European Nucleotide Archive (ENA).

File Name: Supplementary Code 1

Description: R code for reproducing the statistical analysis conducted on the taxonomy outputs from the 12S, 18S and COI gene markers.
