# Supplementary material for: An integrated spatio-temporal view of riverine biodiversity using environmental DNA metabarcoding
Source: Nat Commun. 2024 May 23;15:4372. doi: 10.1038/s41467-024-48640-3 (PMC11116482; doi:10.1038/s41467-024-48640-3)
Supplement: Supplementary file 6 — Reporting Summary [file 41467_2024_48640_MOESM6_ESM.pdf]

nature portfolio

Corresponding author(s): William Bernard Perry, Mathew Seymour, Simon Creer

Last updated by author(s): Apr 16, 2024

Reporting Summary

Nature Portfolio wishes to improve the reproducibility of the work that we publish. This form provides structure for consistency and transparency in reporting. For further information on Nature Portfolio policies, see our Editorial Policies and the Editorial Policy Checklist.

Statistics

For all statistical analyses, confirm that the following items are present in the figure legend, table legend, main text, or Methods section.

n/a	Confirmed	
☐	☒	The exact sample size (*n*) for each experimental group/condition, given as a discrete number and unit of measurement
☐	☒	A statement on whether measurements were taken from distinct samples or whether the same sample was measured repeatedly
☐	☒	The statistical test(s) used AND whether they are one- or two-sided<br>*Only common tests should be described solely by name; describe more complex techniques in the Methods section.*
☐	☒	A description of all covariates tested
☐	☒	A description of any assumptions or corrections, such as tests of normality and adjustment for multiple comparisons
☐	☒	A full description of the statistical parameters including central tendency (e.g. means) or other basic estimates (e.g. regression coefficient) AND variation (e.g. standard deviation) or associated estimates of uncertainty (e.g. confidence intervals)
☐	☒	For null hypothesis testing, the test statistic (e.g. *F*, *t*, *r*) with confidence intervals, effect sizes, degrees of freedom and *P* value noted<br>*Give P values as exact values whenever suitable.*
☒	☐	For Bayesian analysis, information on the choice of priors and Markov chain Monte Carlo settings
☐	☒	For hierarchical and complex designs, identification of the appropriate level for tests and full reporting of outcomes
☐	☒	Estimates of effect sizes (e.g. Cohen's *d*, Pearson's *r*), indicating how they were calculated

Our web collection on statistics for biologists contains articles on many of the points above.

Software and code

Policy information about availability of computer code

Data collection	No software was used.
Data analysis	All statistical analyses were carried out in R v. 3.6.2. R code used in this study can be found in Supplementary Code 1, with no restrictions. The R packages used were 'DADA2' v 1.14.1, 'microDecon' v 1.0.2, 'Vegan' v 2.4.2, 'stats' v 4.1.0, 'mgcv' v 1.8.35 and 'betapart' v 1.6. Other software were also used before the statistical analysis, and these included Cutadapt v 2.3, BLAST+ v 2.10.0 and SILVAngs v 1.9.5/1.4.3 (web front-end/analysis pipeline).

For manuscripts utilizing custom algorithms or software that are central to the research but not yet described in published literature, software must be made available to editors and reviewers. We strongly encourage code deposition in a community repository (e.g. GitHub). See the Nature Portfolio guidelines for submitting code & software for further information.

Data

Policy information about availability of data

All manuscripts must include a data availability statement. This statement should provide the following information, where applicable:

- Accession codes, unique identifiers, or web links for publicly available datasets
- A description of any restrictions on data availability
- For clinical datasets or third party data, please ensure that the statement adheres to our policy

Metabarcoding data used in this study can be accessed from the European Nucleotide Archive under study accession ERP132733. Metadata associated with the samples can be found in Supplementary Data 1. The NCBI nt (https://www.ncbi.nlm.nih.gov/nucleotide/), MitoFish (https://mitofish.aori.u-tokyo.ac.jp/download/),

SILVA r138.1 (https://www.arb-silva.de/documentation/release-138/) and Midori (https://www.reference-midori.info/) databases were also used in this work.

Research involving human participants, their data, or biological material

Policy information about studies with human participants or human data. See also policy information about sex, gender (identity/presentation), and sexual orientation and race, ethnicity and racism.

Reporting on sex and gender	Not been collected.
Reporting on race, ethnicity, or other socially relevant groupings	Not been collected.
Population characteristics	Not been collected.
Recruitment	Not been collected.
Ethics oversight	Not been collected.

Note that full information on the approval of the study protocol must also be provided in the manuscript.

Field-specific reporting

Please select the one below that is the best fit for your research. If you are not sure, read the appropriate sections before making your selection.

☐ Life sciences ☐ Behavioural & social sciences ☒ Ecological, evolutionary & environmental sciences

For a reference copy of the document with all sections, see nature.com/documents/nr-reporting-summary-flat.pdf

Ecological, evolutionary & environmental sciences study design

All studies must disclose on these points even when the disclosure is negative.

Study description

Spatio-temporal eDNA sampling across space (five rivers in Europe and North America, with an upper range of 20-35 km between samples), time (19 timepoints between 2017 and 2018) and environmental conditions (river flow, pH, conductivity, temperature and rainfall). Samples were taken longitudinally along rivers. Three replicate water samples (1L per sample) were collected at each sample site and time point. A total of 939 samples were taken.

Research sample

The research samples were 1L samples of water, from which eDNA was extracted. The rationale for the collection of these samples was to monitor freshwater communities in rivers. These samples are meant to represent populations of riverine metazoans within the vicinity of the sample site, as well as potential transport from populations upstream.

Sampling strategy

Sample sites were arranged in a linear longitudinal transect along the River Conwy (14 sample sites, over a 35.2 km stretch of river in Wales (UK), sampled between 27th April 2017 to 18th April 2018) (figure 1a), River Tywi (12 sample sites, over a 25.7 km stretch of river in Wales (UK), sampled on 13th July 2017), and River Gwash (11 sample sites, over a 27.4 km stretch of river in England (UK), sampled on 31st July 2017), River Glatt (13 sample sites, over a 35.1 km stretch of river in Switzerland, sampled on 3rd July 2017) and Skaneateles Creek, USA (11 sample sites, over a 20 km stretch of river in the USA, sampled on 19th July 2017)(supplementary table 1).

The number of sample sites on the River Conwy, and thus the number of samples per time point, was based on 14 sites, which meant that there was a sample taken approximately every 3.5km of river, taken until the point the river became estuarine. This frequency of sampling along the river was chosen to effectively capture all sections of the river, while also allowing for the transport distance of eDNA to be tested, based on previous estimates of eDNA transport [1,2]. It was this same rational that also informed the chosen number of sample sites in the other rivers.

A total of 19 time points were sampled for each sample site in the River Conwy across a year. The rational for this frequency of temporal sampling was to capture seasonal trends over the year. Sampling occurring, on average, more than once a month across the year which also allowed for a resolution of monitoring beyond seasonal trends, and allowed the capture of important fluctuations in eDNA both within seasons and trends traversing seasons. For the other rivers, samples were all taken in July 2019, to provide a snapshot for cross river comparisons.

1. Jane, S. F. et al. Distance, flow and PCR inhibition: eDNA dynamics in two headwater streams. Mol. Ecol. Resour. 15, 216–227 (2015).
2. Pont, D. et al. Environmental DNA reveals quantitative patterns of fish biodiversity in large rivers despite its downstream transportation. Sci. Reports 2018 81 8, 1–13 (2018).

Data collection

Data was produced using 250 bp paired-end sequencing on an Illumina HiSeq platform aiming for 100,000 reads per sample and target gene (e.g. 12S, 18S and COI). The data was recorded by an Illumina HiSeq platform at the EnviSion, BioSequencing and BioComputing facility at the University of Birmingham (https://www.envision-service.com/).

Timing and spatial scale

Sample sites were arranged in a linear longitudinal transect along the River Conwy (14 sample sites, over a 35.2 km stretch of river in

Wales (UK), sampled between 27th April 2017 to 18th April 2018) (figure 1a), River Tywi (12 sample sites, over a 25.7 km stretch of river in Wales (UK), sampled on 13th July 2017), and River Gwash (11 sample sites, over a 27.4 km stretch of river in England (UK), sampled on 31st July 2017), River Glatt (13 sample sites, over a 35.1 km stretch of river in Switzerland, sampled on 3rd July 2017) and Skaneateles Creek, USA (11 sample sites, over a 20 km stretch of river in the USA, sampled on 19th July 2017)(supplementary table 1). The rationale for the Conwy sampling times was to capture seasonal variations in eDNA. The rational for the sampling times in other rivers was to facilitate comparison.

Data exclusions

Reads within the negative controls were were used to filter readsassociated with samples using the R package microDecon 1.0.2, except for 12S, where the limited number of taxonomic groups meant that the approach was not suitable.The average amplicon size produced by the 12S MiFish primers is 172bp, and so to remove larger bacterial sequences which are also amplified with the 12S primers, amplicon sequence variants (ASVs) that were over 20% longer than this average amplicon size were removed. ASVs were removed if they did not meet a percentage identity of 70%, a query cover of 80% and an e value of 1, with similar cut off parameters having been used previously. Following the broad BLAST, if ASVs were not assigned as bony fish (class Actinopteri), they were removed. Using the ASVs assigned to the class Actinopteri, a second more specific BLAST was conducted on the MitoFish database (downloaded 23rd September 2020), using a higher threshold of 90% percentage identity, 90% query cover and an e value of 0.001, as has been used previously, to ensure accurate taxonomic identification. A curation step was also conducted, whereby ASVs that were assigned taxonomy of a species which were not living in the environment, with the eDNA likely occurring due to secondary introduction (e.g. through human consumption, marine species), were removed (supplementary table 3). ASVs were then clustered into species based on the assigned taxonomy. If a species had less than 0.05% of the overall sample reads it was removed from that sample, if the ASV had less than an absolute value of 20 it was removed and samples containing less than 1,000 reads were also removed. SILVAngs version 1.9.5/1.4.3 (web front-end/analysis pipeline) was used to analyse the 18S sequence data, utilising the SILVA r138.1 database. Using the default settings, the maximum relative amount of ambiguous bases and repeated bases per sequence was set at 2% and 4%, respectively. Using the same threshold as the silva SSU Parc web database, the minimum alignment identity and alignment score of a sequence to a reference sequence was set at 50% and 40%, reBefore the beta diversity analysis, sample outliers, as identified by NMDS plots, were removed (supplementary table 4).spectively. The minimum relative base pair score and minimum relative quality of sequences was set to 30. ASVs were not clustered into Operational Taxonomic Units (OTUs), and so a sequence identity value of 1 was adopted. If an ASV had less than 0.05% of the overall sample reads it was removed from that sample, and samples containing less than 1,000 reads were also removed. Non-metazoans and those metazoans that could not be identified to the phylum level were removed, as well as any sequences that were assigned as primates. A broad BLAST/2.10.0+ search was first conducted against COI gene sequences from the Midori reference database (GenBank release 240), and ASVs were removed if they did not meet an identity of 70% and a query cover of 80%, suggested as a baseline64. If an ASV had less than 0.05% of the overall sample reads it was removed from that sample, and samples containing less than 1,000 reads were also removed. Finally, only metazoan phyla were retained, including: Arthropoda, Gastrotricha, Platyhelminthes, Annelida, Chordata, Rotifera, Mollusca, Cnidaria, Nematoda, Tardigrada, Porifera, Placozoa, Onychophora, Nemertea, Echinodermata and Bryozoa. Due to the high taxonomic resolution of the COI marker, arthropods were further classified into aquatic and terrestrial arthropods based on taxonomy, with only aquatic arthropods kept in the analysis to better assess river biodiversity. Before the beta diversity analysis, sample outliers, as identified by NMDS plots, were removed (supplementary table 4).

Reproducibility

The collection of eDNA samples was replicated across five different rivers. Samples from these different rivers were successfully sequenced, and provided data which could be compared to assess the biodiversity across the respective rivers. Similar trends and patterns in relation to biodiversity were observed between rivers, providing evidence of reproducibility.

Randomization

Samples were allocated into sample sites and time points, which were spatially informed based on the transportation distances of eDNA along a river, and were temporally informed based on the the seasonal change of freshwater biodiversity across a year.

Blinding

Blinding was no relevant during data acquisition because the data was generated using a sequencing platform. Blinding was not relevant for the analyses because a lot of the analysis was exploratory or hypothesis-generating, and when hypotheses were being tested, knowledge of sample information was critical for understanding the context and interpreting results.

Did the study involve field work? ☒ Yes ☐ No

Field work, collection and transport

Field conditions

Sampling was taken over long temporal and spatial scales, therefore, summarizing field conditions here would not be possible. However, metadata included with the manuscript in the supplementary material provides rainfall, temperature and water chemistry for most of the samples.

Location

The five rivers sampled in this study were:
Conwy, Wales (UK)
Tywi, Wales (UK)
Gwash, England (UK)
Glatt, Switzerland
Skaneateles Creek, USA

Again, due to the large spatial scales sampled, summarizing location data is not possible here, however, more detailed location data for each of the 61 sample sites are included in the metadata which is provided in the supplementary materials of the manuscript.

Access & import/export

eDNA sampling is inherently responsible as it is non destructive, and each sample only requires 1L of river water. DNA is then extracted from that water. Therefore, no special access or import/export of samples was required.

Disturbance

eDNA is a non destructive sampling method.

Reporting for specific materials, systems and methods

We require information from authors about some types of materials, experimental systems and methods used in many studies. Here, indicate whether each material, system or method listed is relevant to your study. If you are not sure if a list item applies to your research, read the appropriate section before selecting a response.

Materials & experimental systems

n/a	Involved in the study
☒	☐ Antibodies
☒	☐ Eukaryotic cell lines
☒	☐ Palaeontology and archaeology
☒	☐ Animals and other organisms
☒	☐ Clinical data
☒	☐ Dual use research of concern
☒	☐ Plants

Methods

n/a	Involved in the study
☒	☐ ChIP-seq
☒	☐ Flow cytometry
☒	☐ MRI-based neuroimaging

Plants

Seed stocks	NA
Novel plant genotypes	NA
Authentication	NA
